# *neu*Print: An open access tool for EM connectomics

**DOI:** 10.3389/fninf.2022.896292

**Published:** 2022-07-20

**Authors:** Stephen M. Plaza, Jody Clements, Tom Dolafi, Lowell Umayam, Nicole N. Neubarth, Louis K. Scheffer, Stuart Berg

**Affiliations:** Janelia Research Campus, Howard Hughes Medical Institute, Ashburn, VA, United States

**Keywords:** open science, connectomics, preprint, formal publication, web access, APIs

## Abstract

Due to advances in electron microscopy and deep learning, it is now practical to reconstruct a connectome, a description of neurons and the chemical synapses between them, for significant volumes of neural tissue. Smaller past reconstructions were primarily used by domain experts, could be handled by downloading data, and performance was not a serious problem. But new and much larger reconstructions upend these assumptions. These networks now contain tens of thousands of neurons and tens of millions of connections, with yet larger reconstructions pending, and are of interest to a large community of non-specialists. Allowing other scientists to make use of this data needs more than publication—it requires new tools that are publicly available, easy to use, and efficiently handle large data. We introduce neuPrint to address these data analysis challenges. Neuprint contains two major components—a web interface and programmer APIs. The web interface is designed to allow any scientist worldwide, using only a browser, to quickly ask and answer typical biological queries about a connectome. The neuPrint APIs allow more computer-savvy scientists to make more complex or higher volume queries. NeuPrint also provides features for assessing reconstruction quality. Internally, neuPrint organizes connectome data as a graph stored in a neo4j database. This gives high performance for typical queries, provides access though a public and well documented query language Cypher, and will extend well to future larger connectomics databases. Our experience is also an experiment in open science. We find a significant fraction of the readers of the article proceed to examine the data directly. In our case preprints worked exactly as intended, with data inquiries and PDF downloads starting immediately after pre-print publication, and little affected by formal publication later. From this we deduce that many readers are more interested in our data than in our analysis of our data, suggesting that data-only papers can be well appreciated and that public data release can speed up the propagation of scientific results by many months. We also find that providing, and keeping, the data available for online access imposes substantial additional costs to connectomics research.

## 1. Introduction and motivation

*Drosophila melanogaster* is a well-known model system for studying the structure, function, and operation of the nervous system. One advantage of this system is the large library of genetic lines, each with expression restricted to a subset of cells within the nervous system, and often a single type. This allows individual cell types to be measured, activated, and de-activated, all of which help determine the structure and operation of circuits.

However, genetic access to the cell types alone is not enough to understand circuit operation. Also required is the connections between cells. This was typically obtained by techniques such as GRASP (Feinberg et al., 2008) or trans-Tango (Talay et al., 2017), but these techniques were slow, painstaking, only gave pairwise results, and their accuracy was hard to evaluate. Circuit reconstruction from electron microscope images had the potential to revolutionize the study of circuits by finding all the cell types, and all the chemical synapses between them, for a particular volume of the brain. This potential was demonstrated by early connectomes of columns of the medulla and the alpha lobe of the mushroom body. In these cases, experimental groups such as the Reiser (Strother et al., 2014), Borst (Ammer et al., 2015), and Rubin (Li et al., 2020) labs worked closely with the EM connectome generation groups to get answers to their specific questions about connectivity. These were used to generate hypotheses about circuit operation that were then further investigated using genetic methods.

However, extending connectomes to the bulk of the fly brain introduced new problems of dissemination. Instead of a few groups, the results could now be of interest to thousands of researchers worldwide. This made personal interaction an impractical solution to asking questions about connectivity. Furthermore, the data sets are much larger, painful to download, and the majority of the data is irrelevant to most specific queries. In many ways this is similar to genetic data, where the solution was to keep the data in an on-line database, and queried via a website using algorithms such as BLAST (Altschul et al., 1990) and its successors. However, much more than genetic data (which is one dimensional), connectivity questions take many forms. Since we hoped the typical user would be a biologist, we wanted a web application with the good features of BLAST (no computer science knowledge required, no downloads of programs or data, answers available in a few seconds) but the ability to ask a much wider variety of questions.

Since these requirements could not be met by previous open-access solutions, one of the tasks of the FlyEM project was to build a web interface, where any *Drosophila* biologist world-wide could log on and, with minimal or no training, find answers to common questions about connectivity. NeuPrint, described here, was the result of this effort. Of course, sometimes simple queries do not suffice, and many labs have significant computer science expertise. Therefore, we also built application program interfaces (APIs) to allow automated or bulk downloads of queries and data.

In this paper, we first discuss our overall framework and the main data model. Then, we describe the programmer APIs and web application. Next, we discuss the practical details of deploying neuPrint, and present empirical justification for our design decisions, as well as explore several example queries on a large dataset. Finally, we look at the results of 2 years of experience with this model of open science.

## 2. Methods

High-resolution EM data reveals the morphology of individual neurons and the synapses between them. By representing the neurons as nodes and synapses as edges, the resulting connectivity graph provides scientists one tool to help understand neural mechanisms in brains. Technical hurdles in generating and reconstructing connectomes from EM data limited prior studies to either small brains like *C*. elegans (White et al., 1986) or smaller portions of larger brains (Takemura et al., 2015, 2017; Motta et al., 2019). Despite the relatively small size of individual circuits, typically 1,000 or fewer neurons, compared to the 100, 000 neurons in *Drosophila* or millions of neurons in a mouse brain, deciphering the circuits formed by these neurons is challenging. The need for effective representation of complex connectomes is continually increasing with much larger EM datasets available (Cepelewicz, 2016; Zheng et al., 2018) and the introduction of new methods of speeding up connectomic reconstruction, using techniques such as automatic EM image segmentation using deep learning (Januszewski et al., 2018).

At its simplest, a connectome is a list of each neuron, and its inputs and outputs. In theory, using this connectivity in conjunction with strong genetic tools (such as are available in *Drosophila*), one can selectively silence or monitor specific neurons to potentially infer neural mechanisms for certain behaviors (Serbe et al., 2016). However, even this simple application poses many analysis challenges, especially for larger datasets. If the neuron type being looked up is not well-established and annotated explicitly in the database, how does one find it? Once found, many neurons have hundreds of inputs and outputs spanning large portions of the brain. Which ones are important? The inputs and outputs of even well-known neurons will likely involve brain regions and neurons unknown to the experimenter, or often science as a whole. Very quickly, what seemed like a simple lookup task may require a more complicated analysis, inferring the role of neurons in this population based on their connectivity and projections. The challenges of interpreting large data further intensify if one wishes to infer mechanisms directly from the connectome, such as by trying to find underlying patterns in the connectivity graph or examining low-level motifs such as the location distribution of synapses on a given neuron.

We introduce *neuPrint* as a connectome analysis framework to address the challenges of interpreting large connectome data. At its core, neuPrint is a data model for representing connectome data that provides the following advantages:

- It represents data at different levels of detail based on natural anatomical features (brain region, neuron, connection, and synapse level) to maximize the efficiency of queries based on the needs of the users and to enable an intuitive interface consistent with the goals of the user.
- It exploits a graph database, neo4j (Miller, 2013), a natural fit for connectivity.
- It exploits brain regions (regions of interest, or ROIs) to allow users to take a top-down strategy for understanding complex data. It does this by decomposing connectome data by ROI when relevant.
- It facilitates common and straightforward queries *via* forms and tables, with no programming experience needed. More complex queries are made easier by leveraging the expressive Cypher graph query language.
- It enables metadata properties to be flexibly added to neurons and synapses, and new cellular structures (such as mitochondria) to be added later.

The connectomics data is represented within the graph database neo4j. Over this, we implement an interface expressed in the language of connectomics, allowing users to access the data either programmatically or interactively through a web interface. The neuprint ecosystem optionally links to other storage solutions, e.g., Katz and Plaza (2019), for non-graph connectome-relevant data, such as morphological skeletons, useful in tasks such as delay modeling. The web interface combines 3D visualization and a flexible plugin system to enable the rapid creation of new analysis tools to meet the demands of new usage patterns for this emerging field.

### 2.1. Previous work

There are two relevent areas of prior research. The most similar in terms of user experience is the software used to query genetic information. The most similar in terms of the type of data handled is existing connectome manipulation software.

The previous work that is closest in spirit to the Neuprint web interface are the genetic databases and the query software BLAST and its successors. Genetic queries face many of the same problems as connectomic queries. Both involve very large datasets and queries that typically want to inspect in detail only a small fraction of this data. The solution developed by the genetic community mirrors many of the design decisions here. This includes four key aspects: (a) The data is kept on a server and not downloaded by the individual user. (b) Interaction is through a web interface designed for common queries, all phrased in the language of the subject (in the case of BLAST, sequences). (c) The answer is computed on the server, using methods and data structures optimized for the task. No software needs to be installed, or downloaded on the user's machine. (d) Only the answer is returned to the user.

From the user point of view, this architecture makes the barrier to entry very low. Users can submit queries from minimal systems, or even cell phones, whereas answers can be computed on powerful servers. No downloads or installation of any kind is required. This allows usage on school and corporate computers where software installation is prohibited, and expands the range of potential consumers. The only work required of the user is knowledge of the subject matter.

Other prior works are programs for querying connecomes, which are often tightly bound with programs for creating connectomes (Saalfeld et al., 2009; Beyer et al., 2013; Boergens et al., 2017; Zhao et al., 2018). Between them they provide an impressive collection of analysis tools, an inspiration for many of the functions in Neuprint web and API. This is a difficult area to summarize, as the available programs are complex and rapidly changing, and formal publication tends to lag behind development. However, some of the available software that can be used for querying connectomes is summarized in Table 1.

**Table 1:** Different querying options for connectomes.

**Package**	**Download**	**Database**	**Query language**	**Reports**
CatMaid (Saalfeld et al., 2009)	Yes^a^	PostgrSQL	API calls, forms, SQL	Tables, Graphs, Images, Java data structures
natverse (Bates et al., 2020)	Yes	none	API calls	R data structures
NeuPrint (web)	No	neo4j	Forms, Cypher	Tables, Graphs, Images
NeuPrint (API)	Yes	neo4j	API calls, Cypher	Python data structures
VirtualFlyBrain (Milyaev et al., 2012)	No	PostGres	Forms	Tables, Graphs, Images
Fly Brain Observatory (Lazar et al., 2021)	Yes^b^	OrientDB	Custom (NLP++), SQL-like, Gremlin	Tables, Graphs, Images, Simulations
DotMotif (Matelsky et al., 2021)	Yes	NetworkX, neo4j	Custom Language	Python data structures

*This is an extremely limited summary of a very large field that is constantly changing*.

Notes: ^a^Instances of CatMaid are available on VirtualFlyBrain without downloading at https://catmaid.virtualflybrain.org/.

^b^A subset of queries is available without download at https://www.fruitflybrain.org/#/brainmapsviz.

A wide variety of mechanisms for querying connectomes have been proposed, each with advantages and disadvantages. A natural language interface requires no programming or computer science expertise, but in practice may return unanticipated results or fail to include desired answers. More formal query languages require time to learn, but offer more explicit control of queries. Even among query languages there are differences—path queries in a conventional database query language such as SQL are an advanced topic, but in a graph-specific query language these queries are simpler and more intuitive. Finally, a custom language for a particular purpose (such as searching for motifs as in DotMotif, or selecting cells from multiple sources of information as in NLP++) makes these particular queries simple, at the cost of making different queries more difficult or impossible.

Both the web and API components of neuPrint differ from previous work by using an off-the-shelf database solution, neo4j. Both components also support the most common queries directly, but if the user has a more complex question, they support an open source, well-documented, and reasonably intuitive query language, Cypher.

The web interface to NeuPrint is most similar to “VirtualFlyBrain,” which also needs minimum system requirements, with no download or installation required, and the query internal computation occuring on the server. Compared to “VirtualFlyBrain,” NeuPrint offers many more forms of circuit queries, faster and more general path tracing, and Cypher as a backup for complex queries. “VirtualFlyBrain” supports more complex queries differently, by hosting instances of CatMaid that users can access without download or installation.

The neuPrint API is most similar to “natverse,” though returning results in a different language (Python instead of R), and like the web interface, it offers Cypher as a language for complex queries. Also similar is the NeuroArch python API, which supports SQL-like queries and the graph query language “Gremlin” (Givon et al., 2015).

### 2.2. Storing and representing analysis data

**Figure 1:**
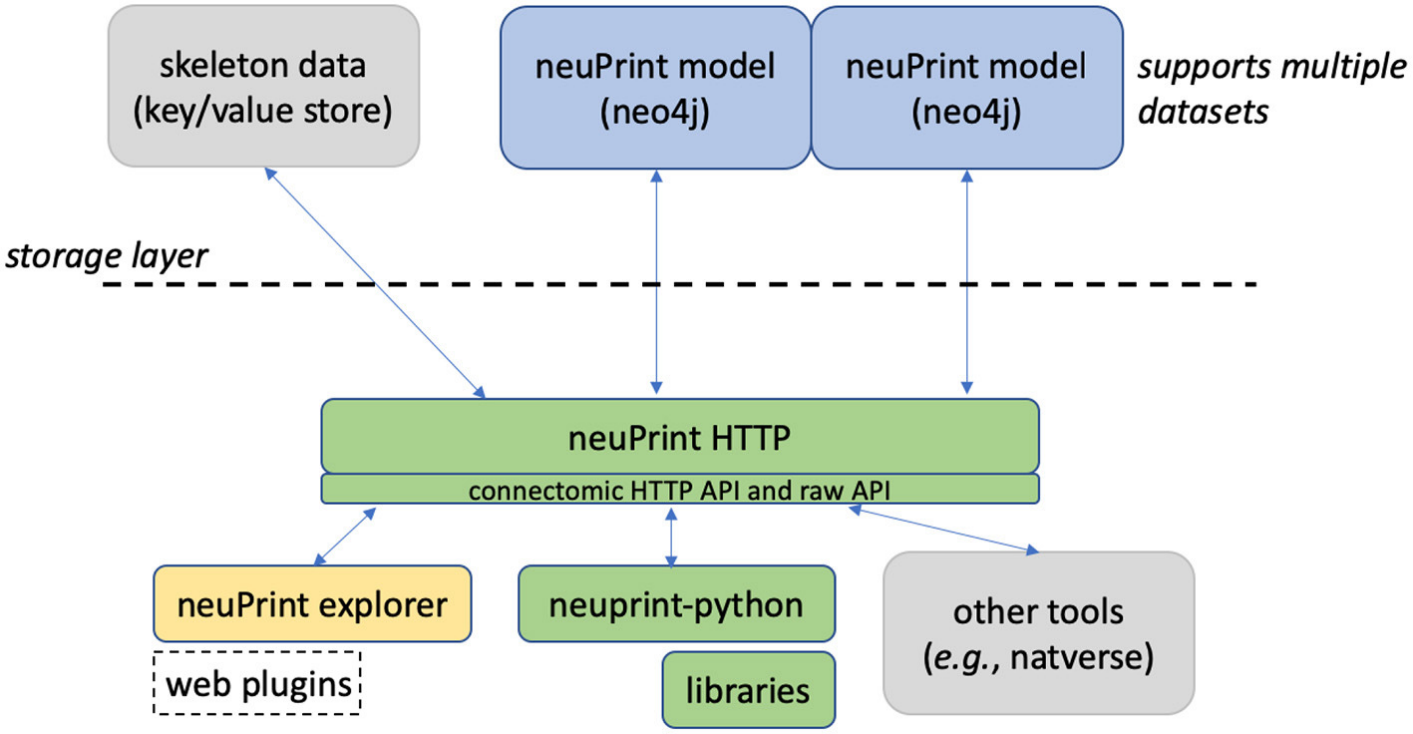
neuPrint ecosystem. The ecosystem is broadly divided into a lower-level data representation and storage above the dashed line and a higher-level interface below the dashed line.

Figure 1 shows an overview of the neuPrint ecosystem. In this section, we emphasize the representation and storage of connectome data. The next section will discuss the higher level interfaces.

We consider the storage of the connectomic graph and associated metadata within a graph database neo4j (Miller, 2013). Presumably, other graph databases that support the graph query language *Cypher* could be compatible with neuPrint, though this has not been tested. In a graph database, nodes can access related nodes through linked lists. This is in contrast to more traditional table-based relational (SQL) databases, where finding whether a node is related to another node requires first joining those two tables together. Therefore, queries that require relationship lookups, such as path searches, are potentially much faster in a graph database.

Graph databases are often advantageous when a data set, and its queries, can be naturally formulated as a graph. A connectome fits this model, with neurons as nodes and synapses as edges. Conversely, in a relational database, a simple graph model showing neuron nodes connected by synapse edges would require several different tables. For instance, one could have a neuron table, a synapse (or edge) table, a neuron property table, and an edge property table. Graph databases and other so-called NoSQL databases tend to not require an exact schema, meaning that it is easy to add new relationship types on pre-existing data models. This is advantageous in connectomics as we anticipate the need to adapt quickly to new analysis requirements, as was demonstrated when we added mitochondria to our data set without disrupting existing access methods.

The EM connectomic dataset involves other data useful for analysis that are not ideally suited for a graph database. For larger storage objects, like a neuronal skeleton (which is a simplified ball and stick representation of a neuron's morphology) and for surface meshes of ROIs, we use simpler key/value stores where one retrieves a value by using a specific key or address. While one can reasonably store a series of skeleton nodes in a graph database, we found that most analyses involving skeletons required the whole skeleton meaning that a simple fetch of the whole data structure was sufficient, and more time and space efficient.

### 2.3. Data model

**Figure 2:**
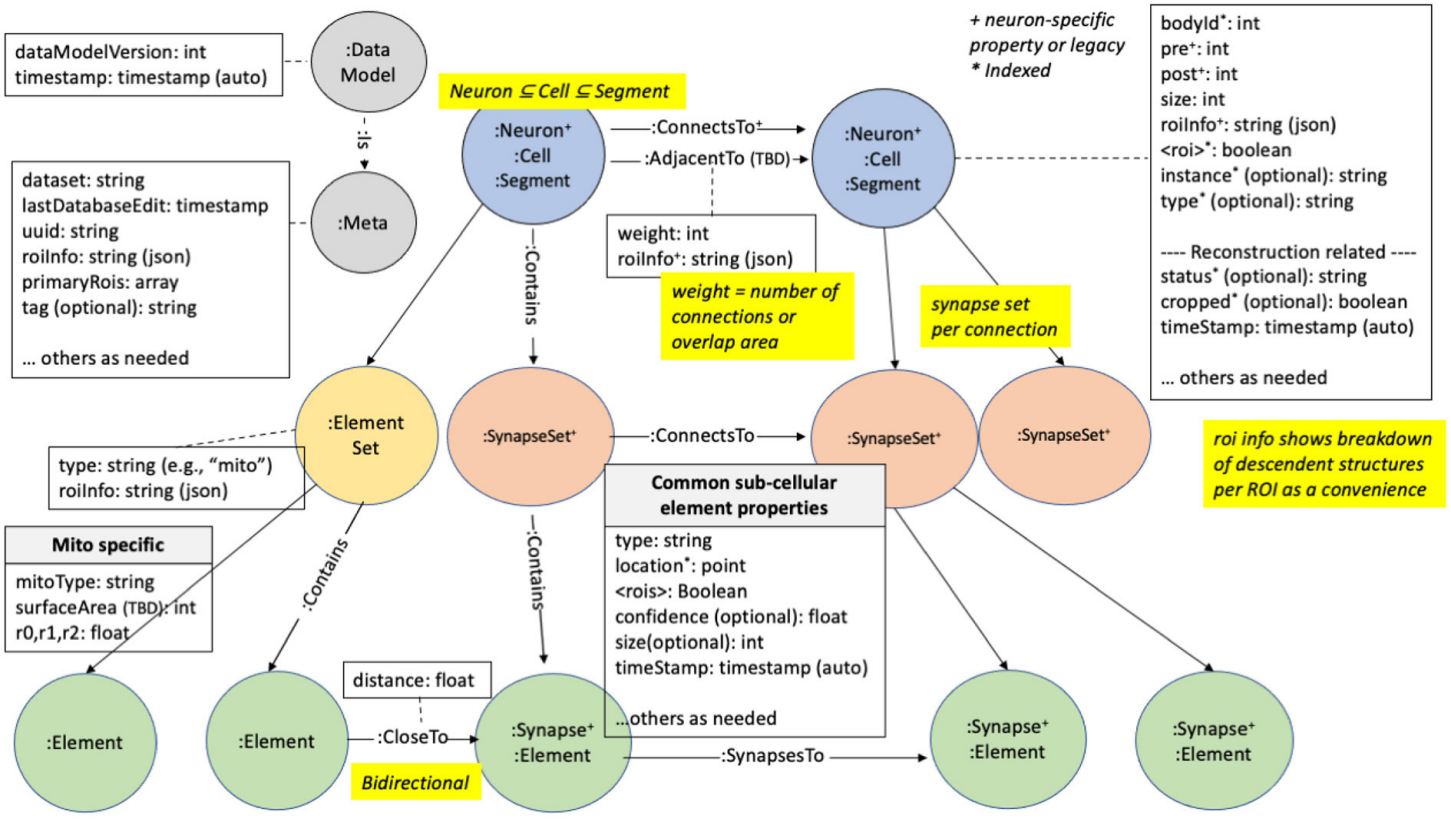
neuPrint graph data model. This shows the various node types and properties used for storing data relevant for connectome analysis.

We illustrate how the data is organized in the graph database in Figure 2. There are five major node types or labels denoted by the syntax “:.” In neo4j these labels help partition the nodes into different groups. :Neuron and :Synapse nodes are two obvious aspects of a connectome. Neurons contain several properties (with more details in the Supplementary Material). The bodyId is a mandatory field and is a unique numerical identifier for a given neuron. Other fields are required as indicated in the figure. neo4j allows indexing of different properties for a node label, reducing querying time at the cost of more disk storage. For example, synapses contain their x, y, and z location, which are indexed properties that can be accessed using neo4j's spatial querying capabilities. The synapses for a given neuron are grouped under different nodes called :SynapseSet. A synapse set groups all the synapses for each connection for each neuron. The :Meta node type provides top-level information about the database.

State of the art techniques (as of 2022) for creating a connectome use automatic image segmentation. This typically does not return complete neurons, instead returning fragments with a variety of sizes, with the small fragments vastly outnumbering the large ones. Each of these fragments is called a :Segment. During the proofreading process (oversimplifying greatly, see Plaza, 2016) smaller segments are merged with larger segments until the largest segments visually resemble neurons. This creates a bi-modal distribution of segment sizes, with a few large one corresponding to neurons, and a much larger number of very small segments with only a few synapses. In theory this could be continued until no small fragments remain, but at the current state of the art this is cost and time prohibitive, and is scientifically unnecessary for the questions most users ask.

Therefore, for several reasons, we explicitly designate larger :Segments as :Neurons. First, the concept of a neuron is critical to the reconstruction process. Only neurons, for example, are given a cell type, an instance name, and a status. Next, the idea of neurons is a critical component of quality metrics. During reconstruction we continually ask ourselves what percentage of all synapses connect two neurons (as opposed to connecting a neuron and a fragment, or two fragments), what percentage of neurons have assigned cell types, and so on. Furthermore, users are almost always concerned with neurons. They typically do not want a query to return a long list of tiny un-named fragments, even if (for example) they are connected to a given neuron. Finally, as neurons are much less numerous than segments, performance is greatly improved on typical searches when they are restricted to neurons.

Between the different node types (segments, neurons, synapses, and synapseSets), we define several relationship types. Prominently, the segments, neurons, and synapse sets are connected *via* a : ConnectsTo property. Individual synapses are linked together through a : SynapsesTo property. We use the : Contains relationship to define the synapse sets that each segment contains and the synapses that each synapse set contains.

Region information is encoded in the data model at multiple levels. Each synapse has a boolean value for each ROI it resides in. Since ROIs are hierarchically defined, several such values may be set. The synaptic ROI information is also aggregated over segments and connections and is stored in the roiInfo field. This enables users to easily extract the number of synapses per region for a segment or connection.

For each node label, we also partition the node using a dataset-specific prefix. For instance, a neuron for the dataset named “x,” would be “:x_Neuron.” In this manner, we can support multiple datasets in the same database. Queries can be made across datasets or targeted to a specific dataset.

Each synapse contains a confidence field, typically computed by automatic synapse prediction, that can be used to model confidence for certain neuron connections.

### 2.4. Design considerations

This section explains the motivation for some of the data model design decisions.

The primary goal of the data model design was to encourage top-down use of the data model and to allow users to exploit region information extensively. The most common queries will only involve neuron connections, which exists as a redundant higher-level representation in our model. An alternative data model design could require the user to extract neuronal connectivity by traversing every synapse between two neurons—a slower, and more complicated query. The ROI information is similarly encoded at multiple levels to facilitate query performance and ease of use. Even though it is possible to compute region statistics from the synapse points, it is faster and easier to find neurons in certain regions and get basic region statistics by simply querying information available at the neuron and neuron connection level.

The current strategy for embedding roiInfo at the connection level and segment level is convenient but clumsy. Because neo4j does not support map datatypes (where a list of keys can have an associated value), the data is encoded as a JSON string. This data cannot be indexed in a meaningful way and requires decoding the JSON when used as a filter within a query. It would also be possible to encode the region breakdown per ROI with the introduction of explicit :Region nodes. While this might be more idiomatic, it leads to more complex user queries, hence our current design decision. Finally, the current data model treats each ROI or brain region separately. If the ROIs available form a hierarchy, one could presumably simplify roiInfo by providing stats only for the ROIs at the lowest level of the hierarchy.

### 2.5. Future considerations

The proposed data model can be extended in many different ways. By allowing multiple datasets in the same neo4j (by using the dataset prefix for each node label), one could add specific relationships between related neurons across datasets. Also, if there are many more property types required for a segment, it might make sense to create a separate :SegmentProperty node. Finally, a relationship type like :Merge could indicate segments that could be grouped together.

Thanks to the schema-less structure of neo4j, we can also extend our model to accommodate other cell ultra-structure without disrupting existing queries and scripts. For example, starting with version 1.2 of the hemibrain data, we have included :Mitochondrion nodes in the connectome, along with links to their nearest other elements.

### 2.6. Interfacing with neuPrint

To enable unified access to the underlying data model and other connectomic data, such as neuron skeletons, we provide a software layer, neuPrintHTTP. neuPrintHTTP is primarily of interest to programmers, not the end users. It provides a mostly read-only connectomic-specific interface that allows users to make HTTP requests that then call the underlying neo4j database or other storage engines. This also simplifies querying within a given dataset. As previously noted, each node label actually encodes the dataset name, such as < dataset>_<node label>. With neuPrintHTTP, the user can direct queries to a given dataset without having to provide dataset-specific labels.

neuPrintHTTP is designed in the language *Go* to exploit convenient concurrency semantics, so it can handle parallel requests efficiently. Furthermore, the backend of the software layer abstracts the storage into different technology-specific plugins. For the non-graph data, plugins exist to access DVID (Katz and Plaza, 2019) and a generic key-value database. Other databases that can satisfy the interface requirements can be easily added, such as Google storage or Amazon S3. neuPrintHTTP also supports authentication with Google OAuth and provides options to make the data read only for anyone, or to restrict access to a set of authorized users. neuPrintHTTP also has a mode to enable database writes for given admin-level authorized users.

As mentioned above, a typical user will not interact with NeuPrintHTTP. Instead they will interact with the web service, or one of the two current APIs that support database access. One is the neuPrint API, written in Python and providing access routines in that language. The other was written by collaborators in Cambridge to provide access to those programming in R (Bates et al., 2022).

### 2.7. neuPrint web explorer

**Figure 3:**
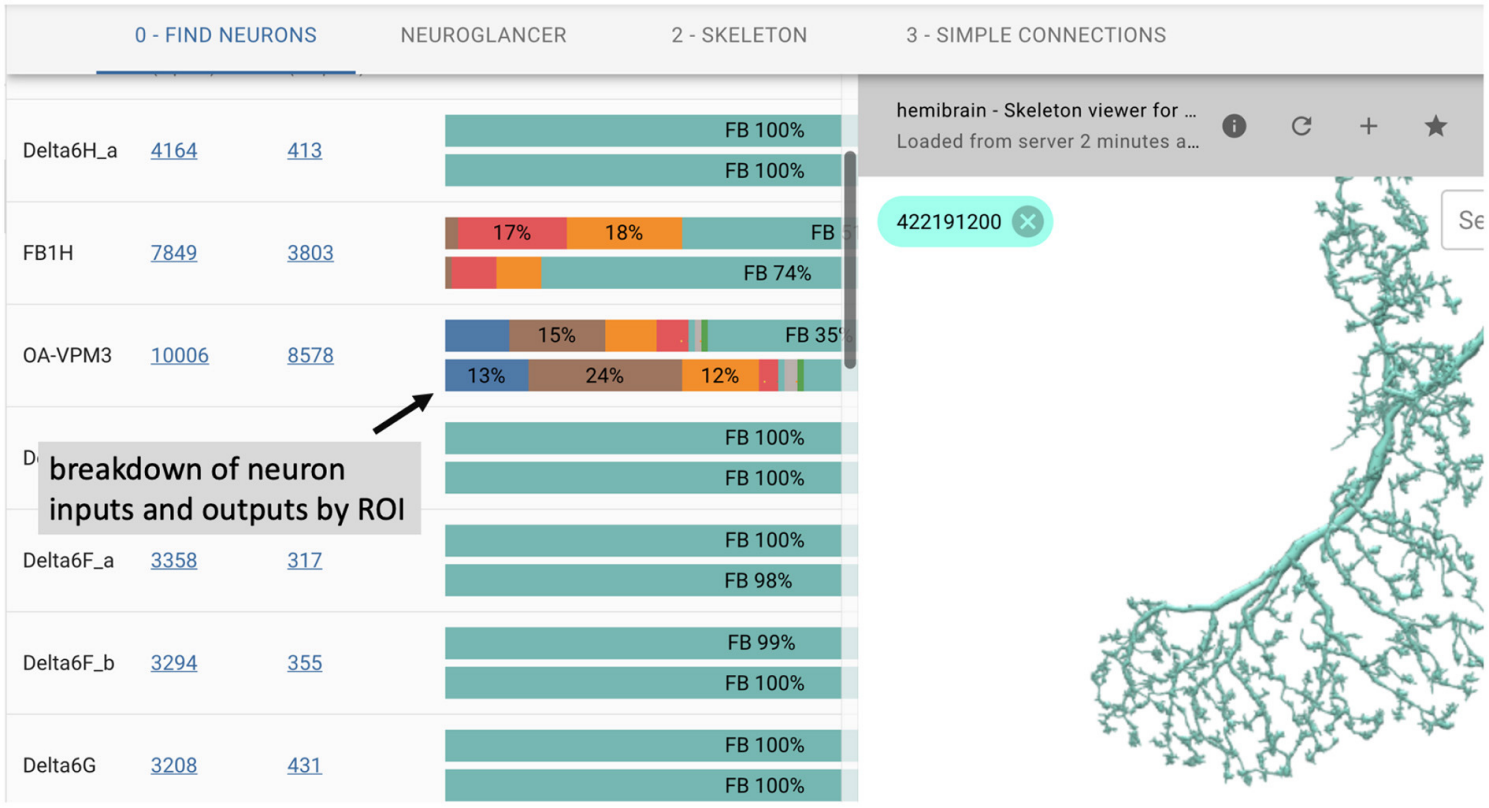
neuPrintExplorer web application. Queries generate tables of results. Visualizations exist to see 3D neurons and to help break down the complexity of the data.

In many cases, users prefer an interactive web-based interface over the use of APIs. To this end, we introduce neuPrintExplorer. neuPrintExplorer is a web application that interacts with neuPrintHTTP, written using the modular web framework called REACT. It provides a series of different common analysis queries within different plugins. Each plugin is a gateway into accessing the data. Internally, most queries involve displaying some table of information based on a simple database request to neuPrintHTTP. In addition, many of these plugins create visuals such as charts that breakdown neuron or connections (see Figure 3) to separate brain regions or provide links to access other parts of the dataset.

As shown in Figure 3, the web application supports 3D visualization of neurons by embedding the skeleton viewing tool called SharkViewer (https://github.com/JaneliaSciComp/SharkViewer). This allows users to see the morphology (shape) of given neurons (fetching the data from the neuprintHTTP's skeleton endpoint) and also the arrangement of synapses on these neurons. We have also implemented a REACT wrapper around the powerful web application neuroglancer (https://github.com/google/neuroglancer), designed for browsing EM datasets. This means that we can embed neuroglancer within our application and enable users to find neurons in neuroglancer based on interactions in neuPrintExplorer. While neuPrint is designed for analysis only, part of the connectome reconstruction process sometimes requires users to add annotations or comments on the underlying dataset. By supporting neuroglancer, neuPrintExplorer provides a gateway for lower-level exploration and annotation if needed.

Architecturally, neuprintExplorer itself is a small application written using REACT/Redux which allows us to leverage other open source components such as d3 for graphics or material-ui for the UI. We also designed the system to be modular by providing a plugin system that allows new queries and views to be added without modifying the core code. There are example plugins and instructions on how to create a new plugin at https://github.com/connectome-neuprint/neuPrintExplorerPlugins.

**Example plugins**

Some of the plugins we have created for the most common tasks are:

- Simple connections: find inputs or outputs for a neuron ordered by connection strength. The data is displayed in table form.
- Find neurons: find neurons in the dataset by name or cell type, optionally restricting queries to specific regions.
- Shortest paths: find all shortest directed paths from one neuron to another and display the local connectivity graph. This query is generally very efficient except for very deep (or non-existent) paths. A timeout is set for a few seconds.
- Find Similar Neurons: find neurons whose inputs and outputs intersect ROIs similar to the provided neuron.
- Cell type: show all neurons of the same cell type to evaluate the connection similarity between neuron of the same type (this is an example of a more complicated query compared to a simple Cypher request).
- Brain region connectivity: show how the brain regions connect to each other by considering the neurons that go from one region to another.
- Common connectivity: view inputs and outputs common to a set of neurons.
- Custom: allow users to execute custom Cypher queries
- Partner completeness (reconstruction QC tools): examine how fragmented the inputs or outputs are for a neuron.
- Completeness (reconstruction QC tools): show the percentage of segments for each brain region that are traced neurons.

To facilitate learning Cypher, relevant plugins display the specific Cypher query made in response to the user query.

### 2.8. Programmer APIs

As mentioned, neuPrint provides an HTTP, or REST, interface to enable programmatic access to the underlying data. Given the diversity of analysis requirements, many of which are currently unknown, we have aimed for a lean HTTP API from which more specific capabilities can be written, such as in our python library or the R packages in natverse (Bates et al., 2020).

The most basic API endpoint provides direct query access to the neo4j interface through the Cypher query language. Cypher shares semantic similarities with SQL and is intended to provide a mostly intuitive language to query a graph database. Below is an example of a query that returns all downstream partners, m, from a given neuron, n with body id 123, with more than 10 connections.

**MATCH** (n :Neuron)-[x:ConnectsTo]->(m)

**WHERE** n.bodyId=123 AND x.weight > 10

**RETURN** m.bodyId, n.instance, n.type, x.weight

Most Cypher queries have these three components. A **MATCH** statement identifies the pattern to be found. In this case, that is a neuron *n* with a connection to *m* (the direction of the connection is indicated by the arrow). A **WHERE** statement applies filters to the above MATCH statement. Here we restrict the match to a neuron *n* with unique body id 123 and with connection weight or strength greater than 10. Finally, the **RETURN** statement provides the results back to the user, which in this case is the ID of the downstream neuron, its name and type, and the weight of the connection. There are several online resources for learning Cypher. We will show a few other examples later in the results section.

In addition to this Cypher interface, neuprintHTTP subdivides its HTTP API into different categories. For example, there is a sub-category called “dbmeta” for database meta information and one called “npexplorer” to provide convenient wrappers for common connectome queries used in the web interface defined below, such as finding neurons that intersect certain regions. This connectomics interface is a work in progress. We plan to extend the interface to provide a simplified wrapper around the most common types of Cypher queries, as access patterns are better understood. More information on this interface can be found at https://github.com/connectome-neuprint/neuPrintHTTP. More information on the python API can be found at https://github.com/connectome-neuprint/neuprint-python.

### 2.9. Quality assessment

Another function of neuPrint is to assess the quality of reconstruction. Here we concentrate on metrics that require the connection graph, as these are hard to do in any other way. First among these are completeness metrics. At the current state of the art, it is too costly and time consuming to connect every synapse to a neuron. So we have added commands that look at a single neuron, or a brain area, and measure what percentage of inputs (or outputs) have synaptic partners that are assigned to labeled neurons (as opposed to those contained in unlabelled fragments). There are also commands that measure what percentage of all synapses in an area belong to traced neurons, and curves of how many segments must be considered to get to a specified percentage of all synapses (pre or post). These commands are useful to both the reconstruction team (to help them decide what most needs improvement) and the end user (who wants to know the completeness of the particular neurons they are studying). These queries are accessible as “Reconstruction Related” under “Change Query Type.”

NeuPrint's reconstruction assessments look only at graph-level completeness metrics. Many other tests are possible and could be useful, such as checking that all synaptic partners are close together, and all synapses on a neuron are close that neuron's skeleton. In our flow, these tests are performed elsewhere and we do not duplicate them here. It would be reasonable to perform these tests on import, to catch any errors that might have slipped through, but as this has not been essential for us we have not yet implemented it.

### 2.10. Initializing, updating, deploying neuPrint

Creating a full neuPrint instance for a new connectomic data set involves ingesting several different types of data. The steps are:

- Create a neo4j database that describes the connectome, organized according to Figure 2.
- Import a skeleton for each neuron, in SWC format (Carnevale and Hines, 2006).
- Import a mesh for each named brain region, in.obj format (LibraryOfCongress, 2020), for rendering in 3D.

Only the neo4j portion is absolutely required.

The primary task involves creating a neo4j database from whatever internal format is used for reconstruction. Importing data into neo4j can be done in many ways as described in documents (neo4j, 2022a,b), tutorials (neo4j, 2022d), and classes (neo4j, 2022c). We ourselves initialize neo4j from a series of CSV files, as documented at https://github.com/connectome-neuprint/neuPrint. The files are formatted to minimize computation in neo4j to speedup ingestion, moving the computational burden to creating these CSV files. We did this because we typically deploy neo4j on a single server, while these CSV files can be generated outside this environment with a compute cluster. We currently only support having one connectome per neo4j database, even though our data model and interfaces allow multiple datasets to share the same database.

The SWC skeleton for each neuron is provided as a file in SWC format. The mesh representation for each brain region is provided as an .obj file. These are POSTed into a DVID instance for use by neuPrint.

The initial ingestion process is streamlined to enable fast, one-time creation of a neo4j instance. As previously mentioned, the neuPrint ecosystem is designed to be compatible with modern connectome reconstruction workflows that allow almost continuous editing. To this end, neuPrint is mostly decoupled from reconstruction workflows except for an incremental interface for updating the underlying data model.

**Figure 4:**
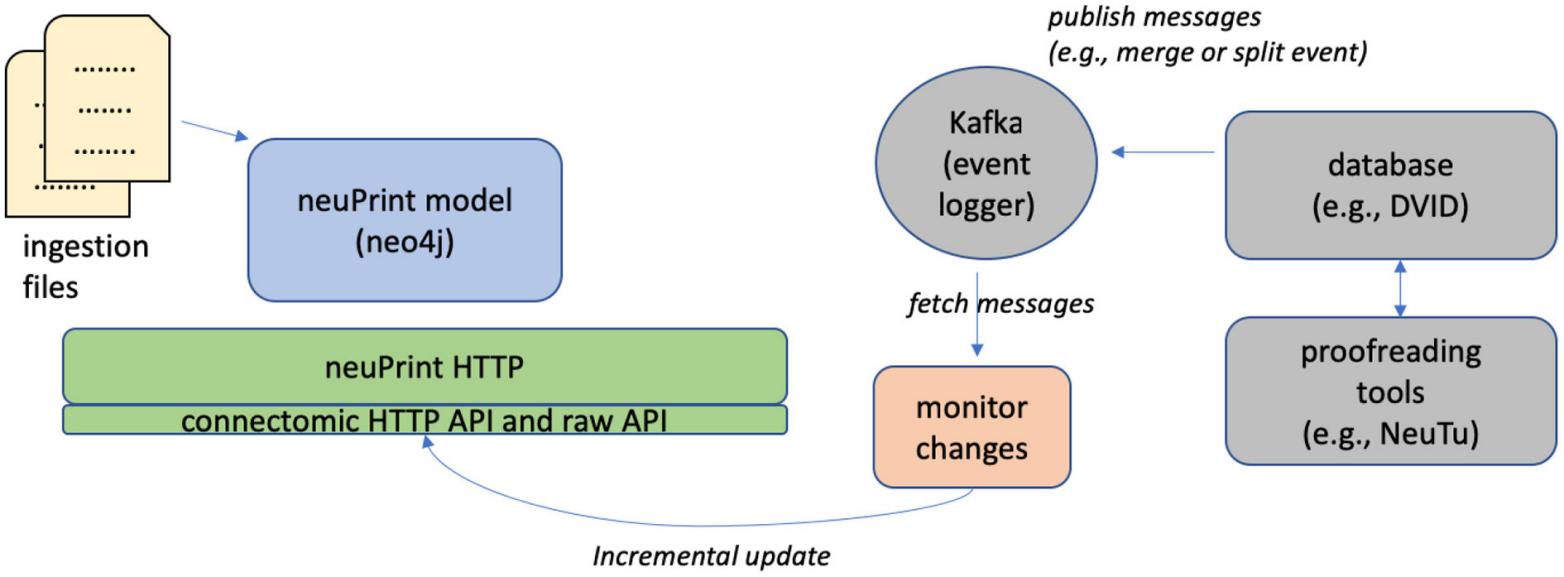
Initializing and updating the neuPrint graph data. The database is initialized by ingesting data *via* a series of CSV files. To update the data incrementally, a service monitors dataset changes recorded in Apache Kafka and makes incremental updates through neuPrintHTTP.

Figure 4 shows the architecture for incrementally updating neuPrint. The key feature is that we require access to only the changes to the dataset, such as segment merge and split events. This can be published by any connectome editor to a centralized log manager, which in our case is Apache Kafka. A monitoring service can listen for changes recorded to this log and modify the neuPrint data model using targeted Cypher statements. For example, a user can modify segmentation data using a tool like *neuTu* (Zhao et al., 2018), which modifies data managed by DVID (Katz and Plaza, 2019). DVID then emits log messages to Kafka, which our Python services then consumes and updates neuPrint graph data through neuPrintHTTP.

Real-time updates have the advantage of always offering the most up-to-date information, but also mean the same query can yield different results when repeated. This can be bad for both debugging and scientific reproducibility, and has only been used internally. For external release we avoid these problems by taking snapshots, which are static and archived as new versions are released. For example, the hemibrain data is now available in versions 1.0.1, 1.1, and 1.2.1. Each is stored in a separate neo4j instance but the user does not see this as it is handled at the neuPrintHTTP level.

## 3. Results (software engineering)

In this section, we provide some insights on the performance characteristics of our system. Comparing neo4j with other relational databases is beyond the scope of this paper. Rather, we try to first demonstrate the effectiveness of our data model and then show that common queries achieve interactive performance (*i.e*. queries are under a few seconds). The example queries explored also serve as documentation for different use cases.

We make available several neuPrint datasets. At https://neuprint-examples.janelia.org we host the fly medulla seven-column dataset (Takemura et al., 2015) and fly mushroom body dataset (Takemura et al., 2017). At https://neuprint.janelia.org we host the hemibrain dataset, about 2/3 of the central brain of a *Drosophila* female with about 20M synapses between traced neurons (Xu et al., 2020). Storage and ingestion performance characteristics are provided in Table 2 for those datasets. The ingestion was performed on a machine with 256GB of memory and 20 processors.

**Table 2:** neuPrint graph representation performance.

**Dataset**	**Ingest (s)**	**Nodes**	**Links**	**Size (GB)**
medulla	8	802K	1,625K	0.15
mushroom body	8	486K	962K	0.09
hemibrain	632	190,746K	369,538K	36

*The ingestion and storage requirements for three example datasets*.

The smaller two datasets, which were some of the largest connectomes produced when they were published, load in only a few seconds. Notably, the much larger hemibrain dataset's load time shows that performance scales well with increased size. As mentioned earlier, we format CSV files to streamline ingestion into neo4j. Notably, the CSV files are about the same size as the neo4j database on disk. The relatively small database sizes for the hemibrain dataset suggest that a even a considerably larger dataset could reside completely within memory on a large server. We expect no problems handling the full fly nervous system (currently in process), as it is less than an order of magnitude larger than the hemibrain and will still sit comfortably within the memory of a single server.

A natural question is whether the advantages of a graph representation will scale to still larger connectomes. The answer is almost surely yes. Even the most speculative connectomes pale in size to existing graph applications in fields such as social media. Consider, for example, a hypothetical mouse connectome (Abbott et al., 2020), a project at least a decade out. The mouse brain is about 508 mm^3^ (Badea et al., 2007), and the synapse density in the cortex is roughly 7.2 × 10^8^ synapses per *mm*^3^ (Schüz and Palm, 1989). Assuming this density holds throughout the brain, this gives a total of about 400 billion synapses, which will determine the overall size of the graph. Social media companies have been handling graphs of similar size for years. Facebook, for example, was processing trillion (10^12^) edge graphs as early as 2015 (Ching et al., 2015). General purpose graph packages have recently scaled to this size as well - in particular, neo4j has already been used on graphs with billions of elements (Fernando et al., 2020), and demoed with 1.2 trillion objects using 240 TB of data, spread across 100 processors (Hunger, 2021). Finally, there is a strong research community extending graph algorithms to ever larger data sets—see efforts such as Reza et al. (2017) and Zhang et al. (2021), among many others. Overall, there is very little doubt that graph handling will be ready when needed by larger connectomic efforts.

For the next two subsections, we evaluate runtime performance of various queries on the hemibrain dataset. The graph data was stored in a cloud VM with memory capacity large enough to hold the entire dataset. Given the remote location of the server, each query includes several milliseconds of latency to access it. All runtime numbers reported are a result of averaging runtimes of over 50 independent queries.

### 3.1. Performance decisions

As discussed in Section 2.4, the graph data model include features that are not strictly needed, but enhance ease of use and runtime efficiency. Three of these decisions were the distinction between segments and neurons, the inclusion of ROIs on connections, and the grouping of synapses into synapseSets. The impact of these decisions was tested through the three different scenarios shown in Table 3. For each scenario, we query the database in two ways: optimized queries that leverage the full data model and less-optimized queries that ignore the redundant information, giving the results we would get with a more simplistic data model. In all cases, the optimized queries are at least 2x faster. While the absolute runtime is relatively fast for two of these scenarios, we find that when the server is exposed to the public, we typically get between 1 and 10 queries per second (see Supplementary Material). Hence a 2x runtime improvement is important in managing the compute load on our servers, and hence the cost of providing this service.

**Table 3:** Optimized vs. non-optimized query performance.

**Query type**	**neuPrint time (ms)**	**No optimization (ms)**
region query (:Neuron vs. :Segment)	480	31,883
ROI info query	27	58
Synapse fetch	42	238

*Examples of different types of queries and the performance in milliseconds of querying using our full data model vs. a simplified subset of the data model*.

**Test 1: Segment vs. neuron**. This test evaluates the decision to partition a subset of :Segment nodes into :Neuron nodes. The motivation was to focus queries on the more important, but less numerous :Neuron nodes. The below queries count the number of neurons or segments over a certain size in each region.

Counting neurons for an ROI.

**MATCH** (n :Neuron) **WHERE** n.ROI AND n.size > 100000000 **RETURN** count(n)

Counting segments for an ROI.

**MATCH** (n :Segment) **WHERE** n.ROI AND n.size > 100000000

**RETURN** count(n)

In this example, the large performance disparity is also due to ROI names being indexed to :Neuron. But even if we force a linear scan through all :Neuron nodes (which involves 1/100 the number of total segments), we still observe queries under 1 s. We could in principle create indices for every property for a segment, but each index comes with a storage cost which is magnified because there are many more segments than actual neurons. Therefore, having a special :Neuron designation potentially reduces the database size and can improve performance.

**Test 2: roiInfo**. This test checks the performance of using the :ConnectsTo property, roiInfo, vs. examining the ROI information by inspecting individual synapses. We constructed two queries examine a given connection to see if the connection is in a given ROI. The first uses the roiInfo property on the connection edge. The second one inspects region information by looking at all the synapses within a synapse set. The detailed queries are specified in the Supplemental Material. We find that using the roiInfo property results in a 2x faster query, and more importantly, the query is much more compact and easier to understand.

**Test 3: Accessing synapses through synapse sets**. This test examines our decision to use :SynapseSets as a mechanism of grouping synapses together. In general, by grouping synapses together, we can minimize the number of edges on a given :Segment in the graph model, presumably accelerating queries involving segments. In our model, :SynapseSet nodes are specific to each connection between two segments. We compare queries that download all synapses for a given connection either using synapse sets or by determining the relationships by exploring the lowest level :SynapsesTo relationship.

Both Cypher queries are relatively complex to express. However, the synapse sets enable much faster performance.

### 3.2. Example queries

In this section, we survey different analysis use cases and provide a sense of runtime performance averaged over several runs, as shown in Table 4. Notably, most queries require only a fraction of a second. For the two queries that involve a single neuron (“connections to traced neurons” and “partner completeness”), running the same command on the largest neuron (“APL,” more than 100K synapses) resulted in a time only twice the average. A wider distribution was seen for the most complex query, involving looking for all 3-hop paths between several random pairs of neurons. The average runtime for this query is under 5 s but we noted a wide variance with many queries finishing under a second and some taking around 30 s. Again, the specific Cypher queries are given in the Supplementary Material.

**Table 4:** Performance of example queries.

**Query type**	**Runtime (ms)**
Connections to traced neurons	27
Path search	4,840
ROI projection	487
Reciprocal connection	27
Partner completeness	29
Name search	46

*Runtime in milliseconds is averaged over several different queries of the same type*.

Example 1: sum the connection weight of a specified neuron to all partners with status “traced.”

Example 2: find all paths up to length 3 between two neurons, where all connections have weight >=5.

Example 3: Count the neurons projecting from one region to another.

Example 4: Find if a reciprocal connections exist between a pair of neurons.

Example 5: Find reconstruction incompleteness for a neuron's outputs (returns the distribution of reconstruction statuses).

Example 6: Count the neurons whose types match a regular expressions.

## 4. Results (open access)

Now that NeuPrint has been available for almost 2 years, we can look back and see how open access to the connectome is working. For this project, we published the data alone in January 2020, without any significant analysis. This involved a bioRχivpaper (Xu et al., 2020) and making the web site and APIs available to the public for queries. About 8 months later, we published a more polished paper in eLife (Scheffer et al., 2020) and an updated dataset on the web. The delay was for several reasons. The first was the time required by peer review and the resulting responses and corrections. The second was to include some analysis, as it was thought that no biology journal would accept a paper with only data and no analysis of that data. The final reason was self-imposed (and the longest in terms of time). By the time of the pre-print, we had assigned names and types to all neurons in the commonly researched areas of the brain, where knowledge and experts are plentiful. Thus, the pre-print, and the initial data, covered almost all of the questions asked by existing researchers. But for the formal release, we wanted consistent naming and typing of *all* the neurons in the hemibrain, so when these were studied in the future, we could avoid name conflicts and inconsistent notation. This was a very worthwhile exercise, but its benefits were in the future, not the present. There was no benefit to immediate, practical questions, since the changes were limited to the largely unstudied region of the brain, and the existing researchers did not need our analysis. We suspect this is common in scientific research, where the data is useful well before it is perfect and fully analyzed. In such cases releasing the data well before publication can speed up progress in the field as a whole.

Since the initial release, we have tracked several metrics to see how well the open access to both the data and publications has worked. For this analysis, we used readership and download data from both bioRχivand eLife, citation data from Google scholar, and on-line access statistics from our internal web server logs. These web access logs have significant limitations. They were designed to support our software engineering, and not intended to support open-access monitoring. The logs are from a single machine, while several were supporting queries. This does probably not affect the count of users much, as the typical user submits many queries during a session and most likely at least one query would appear on each machine running at the time. It does mean the query counts are low, likely by a factor of at least two. Some queries (such as those containing improperly escaped characters) are not handled correctly by our logging software, and were ignored here. There is a gap in early 2021 where the some logs were accidentally deleted. Despite these problems, the data is believed to fairly represent the actual usage.

**Figure 5:**
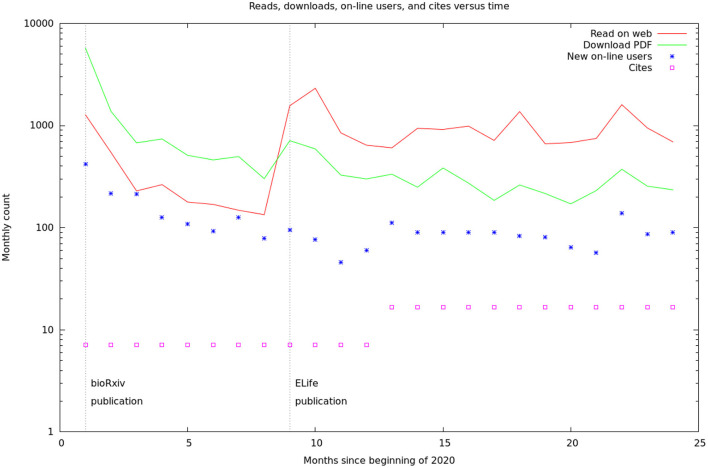
Relative rates of readership, database access, and cites. The readership and download rates are the sum of the figures from the bioRχivand eLifesites. The database access rates are from our logs. The citation rates are from Google scholar, but are only shown yearly and have been converted to monthly rates for this graph. The vertical lines are the initial BioRxiv publication (Xu et al., 2020) and the formal, peer-reviewed eLifearticle (Scheffer et al., 2020).

**Figure 6:**
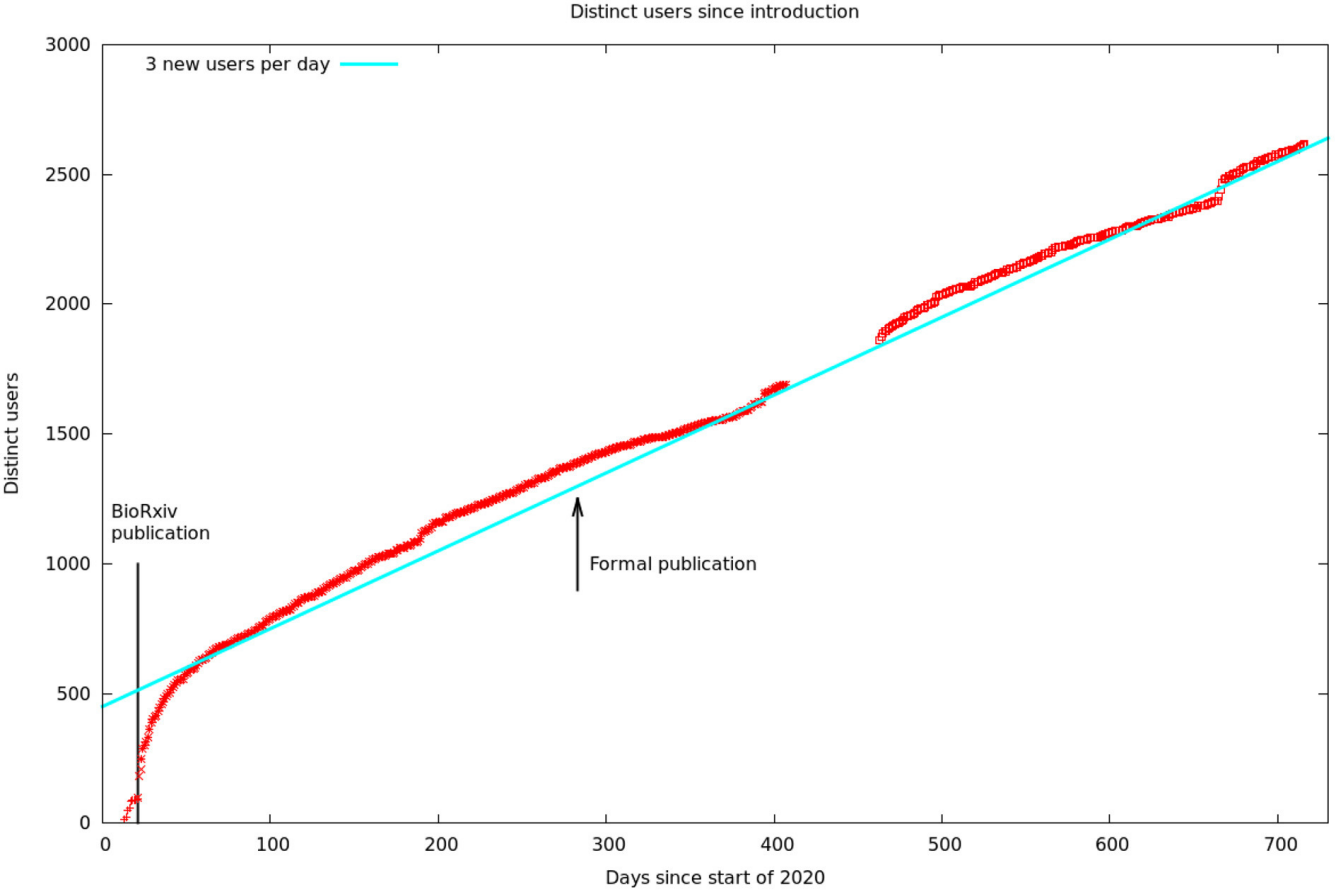
Cumulative unique visitors. The solid line is a hypothetical rate of three new users per day. We assumed this rate during our data gap, as this is consistent with the rest of our data. The solid line to the left is the date of publication of the first bioRχivpaper. The arrow shows the later formal publication in eLifethat contained better cell typing, analysis, and discussion.

These data are summarized by month in Figure 5. It would appear there are two different audiences for our papers and data. One group, likely people already working in the field, started downloading the PDF of the article, and logging onto the web site, as soon as the bioRχivpaper was released. These user's behavior seems largely unaffected by the release of the formal article, a conclusion supported by the more detailed log statistics in Figure 6. On the other hand, formal publication increased the number of readers who read the article on line, but not the number of downloads or logins. We suspect this group of scientists who are interested, but not doing research in the area.

Figure 6 shows the number of unique visitors over the 700 days the data has been available.

As mentioned above, it is “tribal knowledge” that data alone is insufficient for a prestige journal publication. One implication is that scientists who work full time to generate excellent data (as opposed to analyzing it) do not get full credit for their contributions. Our results show that data alone can indeed make a significant contribution, and that data generation, as well as data analysis, perhaps deserves consideration by major journals.

Overall, it would appear that in this case, the scientific usage of our results was advanced by about 8 months by publishing the data as soon as the most commonly used subset was solid, and not waiting for full completion, analysis, and journal publication. Similar conclusions has been reached about the effect of preprints and rapid data release on the progress of research into COVID-19 (Watson, 2022). Some scientists have worried that publishing the raw data early would enable others to examine the raw data, cherry-pick the easiest and most sexy results, and then rush them into publication and hence steal credit from those that did the hard work. This did not happen in our case.

Some interesting statistics from our experience (see Supplementary Material): On an average day, 30 people will read the article on-line, 10 will download the PDF, and 60 will log onto the web site. Of the 60 who logged on, 57 are repeat customers and 3 are new. All these reads and queries will result in about 0.5 cites.

The time constant to steady state after the initial bioRχivrelease was about 13 days (see Supplementary Material). Roughly 30% of *Drosophila* scientists work on weekends. The rate of queries to the database is often more than one per second, and sometimes more than 10 per second, so efficiency optimizations were well worthwhile.

A non-scientific issue is the cost of maintaining open access. There is no public repository for connectomic data, so researchers in the field, and some journals, have been pressing for a pledge to keep the on-line resources available for at least 10 years. Currently, the neuPrint server is a $50,000 machine sitting outside our institutional firewall. If we needed to purchase an equivalent service from a cloud provider, we estimate the cost at about $5,000 per year, for a similar total cost. Another expense is the storage needed for the data, particularly the gray-scale data which is about 7TB. For now, Google is bearing this cost as a part of our cooperation in connectomics. If we needed to pay for this, it would be another $2,000 per year. In addition to these costs, there is the cost of software maintenance. Even though we are not changing the published data at this point, things can and do still go wrong as compute infrastructures evolve. This cost is hard to estimate, particularly for future potential changes, but is likely comparable to the other expenses mentioned. Overall, therefore, we estimate the cost of providing open-access to the data over a 10 year span to be roughly $100–200K. This is a non-negligible sum for all except the largest projects.

Based on our results, we now believe the time is right for a centralized connectome data bank, as the genetic community has in GenBank (Benson et al., 2018). This would likely be funded through the BRAIN initiative (Mott et al., 2018) or perhaps its global extension (Yuste and Bargmann, 2017). This would address two major problems facing the field. First, there is currently no common format, or common access method, for accessing the connectomes of different groups. A centralized repository would by necessity make this effort a priority. This takes on even more significance as comparing connectomes will become its own field as soon as enough connectomes are available. Second, this would solve the problem of connectomic data becoming inaccessible as people and institutions move on. Right now, keeping connectomics data online depends on the good will, and funding, of the scientists who did the original research. This is not a sustainable model.

## 5. Conclusions

We introduce the neuPrint ecosystem in this paper as a mechanism to aid in large-scale analysis of EM connectomes. The central component of neuPrint is the graph data model that stores the data in an efficient manner, accessible to a variety of users and use cases. To this end, we highlight both a custom interactive web interface and programmer interfaces. Our results show that our database enables a diverse set of queries with a dataset containing millions of synaptic connections.

Creating platforms and resources for large EM connectomic datasets pose different challenges than other neuroscience resources, such as VirtualFlyBrain (Milyaev et al., 2012), the Allen Brain Map (Sunkin et al., 2012), or the Mouse Light project (Winnubst et al., 2019). These resources typically involve the collection of several (often smaller) datasets that are combined to form canonical atlases. In the case of connectomes, a single dataset is expensive to acquire and often very large. The notion of a canonical connectome atlas is less meaningful currently. As such, neuPrint emphasizes access to specific datasets rather than a general compilation of many datasets.

An EM image volume often contains much more information than simply neurons and synapses. Future work will involve incorporating information about the location and arrangement of various sub-cellular organelles into the data model, as we have already done for mitochondria. We believe that tools like neuPrint will be critical for managing the complexity of such rich datasets, especially as the means for extracting this information automatically become more reliable.

In terms of open science, we find that a bioRχivrelease and internet access to the data reaches a subset of practitioners in our field several months earlier than formal publication, and for this subset formal publication has little additional effect. Formal publication, however, does reaches more readers in general. We find a significant fraction of the readers of the article proceed to examine the data directly. We also find that providing, and keeping, the data on line impose a substantial additional cost to connectomics research.

## Data availability statement

The datasets presented in this study can be found in online repositories. The names of the repository/repositories and accession number(s) can be found below: https://neuprint-examples.janelia.org; https://neuprint.janelia.org; https://github.com/JaneliaSciComp/SharkViewer; https://github.com/google/neuroglancer; https://github.com/connectome-neuprint/neuPrintExplorerPlugins; https://github.com/connectome-neuprint/neuPrintHTTP; https://github.com/connectome-neuprint/neuprint-python; https://github.com/connectome-neuprint/neuPrint; https://natverse.org/neuprintr; https://doi.org/10.5281/zenodo.6842208.

## Author contributions

SP developed the conceptual design, early versions of the software and this paper. JC implemented the front end (user facing) part of the interface. TD implemented our instances of the neo4j database. LU did the programming to convert from our internal formats to the formats needed for open access, NN built the initial version of the web interface, LS analyzed the usage data, used neuPrint for published analyses, provided user feedback and wrote the final paper. SB wrote the python API and managed the project. All authors contributed to the article and approved the submitted version.

## Funding

Funding for this research was supplied by the Howard Hughes Medical Institute.

## Conflict of interest

The authors declare that the research was conducted in the absence of any commercial or financial relationships that could be construed as a potential conflict of interest.

## Publisher's note

All claims expressed in this article are solely those of the authors and do not necessarily represent those of their affiliated organizations, or those of the publisher, the editors and the reviewers. Any product that may be evaluated in this article, or claim that may be made by its manufacturer, is not guaranteed or endorsed by the publisher.
