# Supplementary material for: neuPrint: An open access tool for EM connectomics
Source: Front Neuroinform. 2022 Jul 20;16:896292. doi: 10.3389/fninf.2022.896292 (PMC9350508; doi:10.3389/fninf.2022.896292)
Supplement: Supplementary file 1 [file Data_Sheet_1.PDF]

Supplementary Material: *neu*Print: An Open Access Tool for EM Connectomics

1 SUPPLEMENTARY MATERIAL AND DATA

This section defines many of the various properties defined on the neo4j data structures.

:Segment (:Neuron) nodes

- bodyId: a unique number for each distinct segment
- pre: Number of pre-synaptic sites on the segment
- post: Number of post-synaptic sites on the segment
- type: Cell type name for given neuron (if provided)
- instance: String identifier for a neuron (if provided)
- size: Number of 8x8x8 nm voxels in the body
- roiInfo: JSON string showing the `pre` and `post` breakdown for each ROI the neuron intersects.
- <roi>: This property only exists for the ROIs that intersect this segment
- status: Reconstruction status for a neuron. By convention, we broadly consider proofread neurons as being "Traced".
- cropped: Boolean describing whether a significant portion of the neuron extends outside the reconstructed volume. By convention, all "Traced" neurons have an explicit value.

:Synapse nodes

- confidence: floating point value between 0 and 1, representing the confidence of the synapse annotation (higher score means more confidence).
- location: x,y,z location for the synapse. This location is always inside its parent segment. The location is indexed to enable fast spatial queries.
- <roi>: This property only exists for the ROIs that intersect this synapse

:ConnectsTo relationship (between two :Segment nodes)

- weight: number of synapses (or weight) between the two neurons
- roiInfo: JSON string showing the `pre` and `post` breakdown for each ROI the connection intersects

:Meta node

- uuid: some version for this dataset. This could be a DOI. Similar to a GIT commit ID used in software development.
- tag: a release tag similar to the tags provided for software releases (e.g., "v1.0")
- primaryRois: an array of ROIs that make up the primary ROIs (or default level of ROIs in the ROI hierarchy). This is useful for various plugins in neuPrint explorer.

- roiInfo: JSON string showing the `pre` and `post` breakdown for each ROI.

1.1 Supplemental data on NeuPrint usage

This is data we found interesting, but overly specific for the main paper. Figure S1 shows the number of unique vistors per day who were motivated enough to sign in (users who are just browsing, without signing in, do not count).

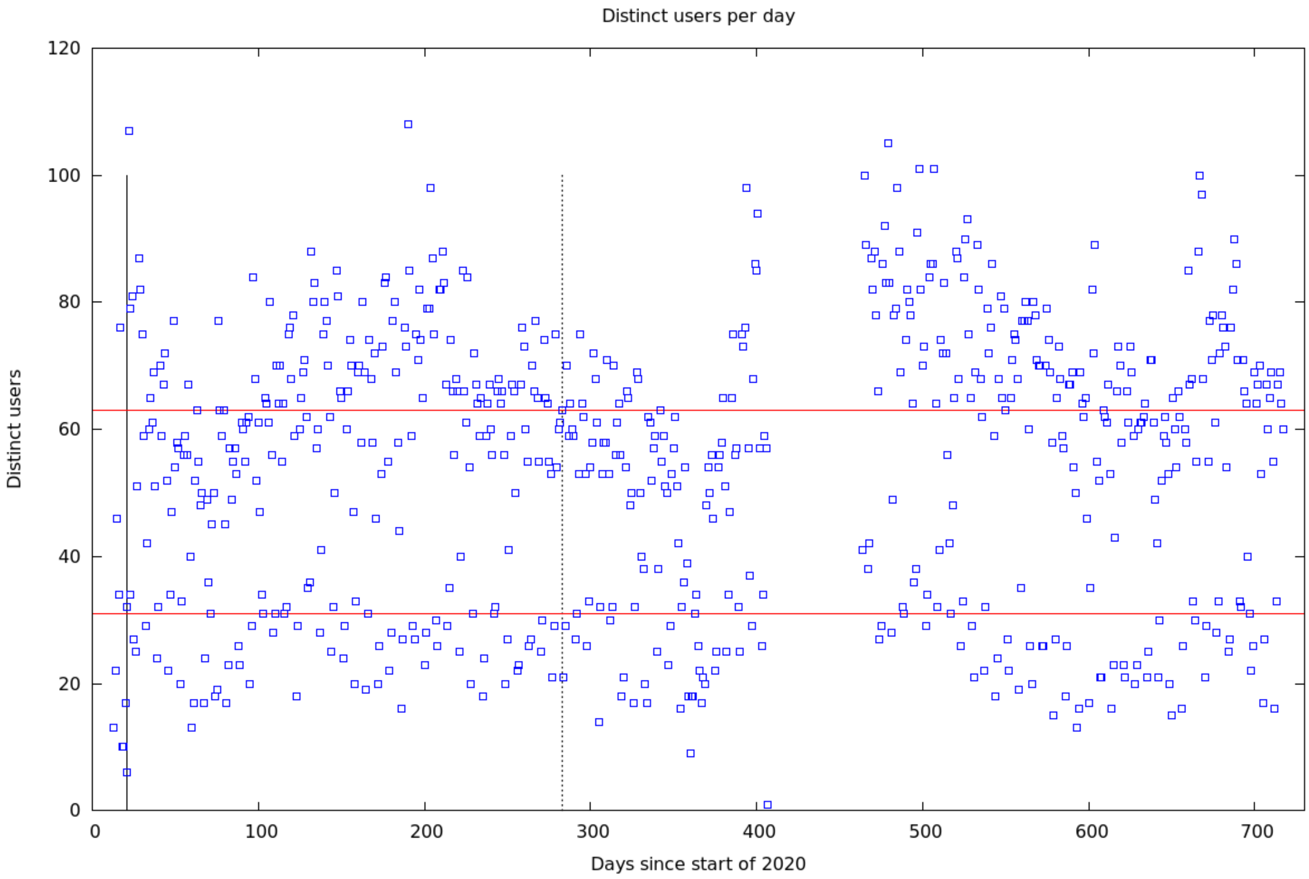

Figure S1. Unique visitors per day. The solid vertical line is the date of publication of the first bioRxiv paper(Xu et al., 2020). The dotted vertical line is the later formal paper Elife(Scheffer et al., 2020) that contained better cell typing, analysis, and discussion. The data appears in two bands - the top band are weekday accesses, the lower band weekend. The solid horizontal lines are the averages for each, computed as described below.

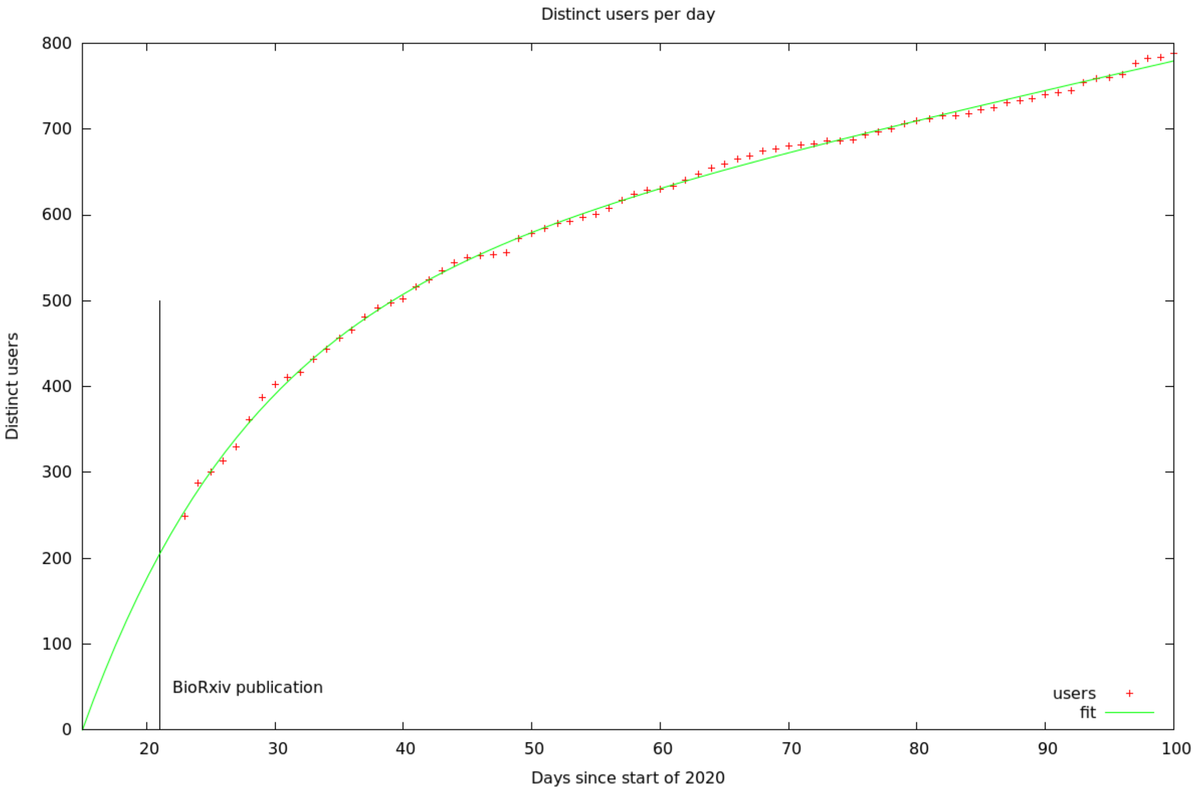

Figure S2. Adoption rate of neuprint. This plot shows the number of unique users per day, for the first 80 days after the publication of the BioRxiv articleXu et al. (2020) describing the connectome. The solid line is a linear + exponential fit to this data. The time constant for the exponential is 13 days. The solid vertical line is the date of publication of the first bioRxiv paper.

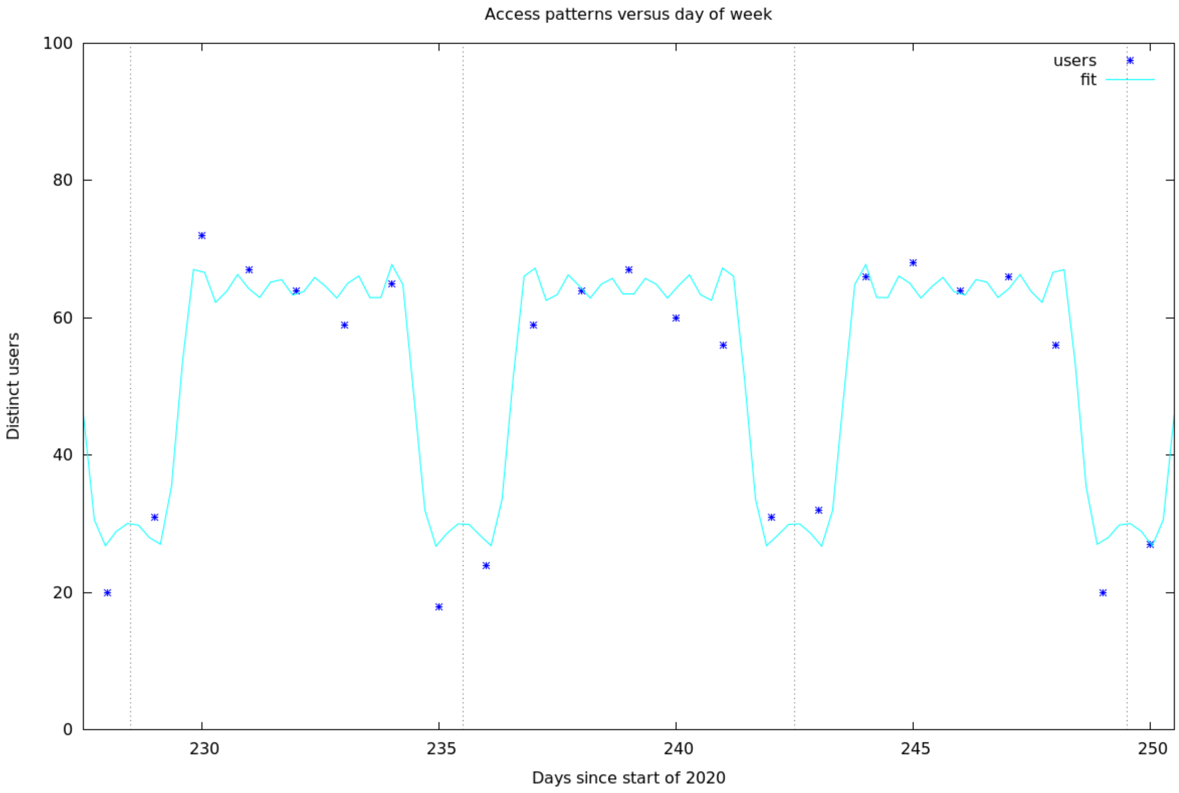

Figure S3. Usage versus day of week. This plot shows the number of unique users per day, for three weeks during the first year of publication. Vertical dotted lines are week boundaries. The solid line is a fit of an eight-harmonic approximation of a 2:5 ratio on/off waveform to the entire post-publication access data (not just the three weeks shown)..

2 DETAILED QUERIES

Test 1: Segment vs neuron. This test evaluates the decision to partition a subset of `:Segment` nodes into `:Neuron` nodes. The motivation was to focus queries on the more important, but less numerous `:Neuron` nodes. The below queries try to count the number of neurons or segments over a certain size in each region.

Counting neurons for an `ROI`.
MATCH (n :Neuron) **WHERE** n.`ROI` AND n.size > 100000000 **RETURN** count(n)

Counting segments for an `ROI`.
MATCH (n :Segment) **WHERE** n.`ROI` AND n.size > 100000000 **RETURN** count(n)

In this example, the large performance disparity is also due to ROI names being indexed to `:Neuron`. But even if we force a linear scan through all `:Neuron` nodes (which involves $1/100$ the number of total segments), we still observe queries under one second. We could in principle create indices for every property for a segment, but each index comes with a storage cost which is magnified because there are many more segments than actual neurons. Therefore having a special `:Neuron` designation potentially reduces the database size and can improve performance.

Test 2: roiInfo. This test checks the performance of using the `:ConnectsTo` property, `roiInfo`, versus examining the ROI information by inspecting individual synapses. The following two queries examine a given neuron connection to see if the connection is in a given ROI. The first uses the `roiInfo` property on the connection edge. The second one inspects region information by looking at all the synapses within a synapse set.

Checks if connections exist between `body1` and `body2` in a certain `ROI`.
MATCH (n :Neuron {bodyId: `body1`})-[x :ConnectsTo]->(m :Neuron {bodyId: `body2`})
RETURN EXISTS(apoc.convert.fromJsonMap(x.roiInfo)["`ROI`"])

Checks if connections exist `body1` and `body2` in a certain `ROI` without using denormalized roi information.
MATCH (n :Neuron {bodyId: `body1`})-[:Contains]->(:SynapseSet)-[:ConnectsTo]
->(ss :SynapseSet)<-[:Contains]-(m :Neuron {bodyId: `body2`})
MATCH (ss)-[:Contains]->(syn :Synapse)
WHERE syn.`ROI`
RETURN true LIMIT 1

While using the `roiInfo` property results in a 2x faster query, more importantly, the first query is much more compact and easier to understand.

Test 3: Accessing synapses through synapse sets. This test motivates `:SynapseSets` as a mechanism of grouping synapses together. In general, by grouping synapses together, we can minimize the number of edges on a given :Segment in the graph model, presumably accelerating queries involving segments. In our model, `:SynapseSet` nodes are specific to each connection between two segments. The queries below

provides an example of downloading all synapses for a given connection either using synapse sets or by determining the relationships by exploring the lowest level `:SynapsesTo` relationship.

Retrieving post-synaptic sites for connections with `:SynapseSet`.
MATCH (n :Neuron {bodyId: `body1` })-[:Contains]->(:SynapseSet)-[:ConnectsTo] ->(ss :SynapseSet)<-[:Contains]-(m :Neuron {bodyId: `body2` })
MATCH (ss)-[:Contains]->(syn :Synapse)
RETURN syn.location, syn.confidence

Retrieving post-synaptic sites for connections without `:SynapseSet`.
MATCH (n :Neuron {bodyId: `body1`})-[:Contains]->(:SynapseSet)
-[:Contains]->(:Synapse)-[:SynapsesTo]->(syn :Synapse)<-[:Contains]
-(ss :SynapseSet)<-[:Contains]-(m :Neuron {bodyId: `body2`})
RETURN syn.location, syn.confidence

2.1 Performance

Example 1: sum connection weight of partners that are traced.
MATCH (n :Neuron {bodyId: `bodyid`})-[x :ConnectsTo]->(m :Neuron)
WHERE m.status="Traced"
RETURN sum(x.weight)

Example 2: find all paths up to 3 in length between two neurons with >=5 connections.
MATCH p =(n :Neuron { bodyId: `body1` })-[x :ConnectsTo*..3]
->(m :Neuron bodyId: *body2*
WHERE ALL (x in relationships(p) WHERE x.weight >= 5)
RETURN count(p)

Example 3: find neurons projecting from one region to another.
MATCH (n :Neuron)
WHERE n.`ROI1` AND n.`ROI2`
WITH apoc.convert.fromJsonMap(n.roiInfo) AS info, n
WHERE info[`ROI1`].post > 0 AND info[`ROI2`].pre > 0
RETURN count(n)

Example 4: Find reciprocal connections.
MATCH (n :Neuron {bodyId: `body1` })-[x :ConnectsTo]
->(m :Neuron {bodyId: `body2` })
WHERE (m)-[:ConnectsTo]->(n)
RETURN true

Example 5: Find reconstruction incompleteness for a neuron's outputs (returns the distribution of reconstruction statuses including which connections are "Traced").

MATCH (n :Neuron { bodyId: *bodyid* })-[x :ConnectsTo]->(m :Segment)
RETURN m.status as status, sum(x.weight) AS total

Example 6: Lookup neuron type with regular expressions.
MATCH (n :Neuron)
WHERE n.type=∼"*prefix*.*"
RETURN count(n)

REFERENCES

Scheffer, L. K., Xu, C. S., Januszewski, M., Lu, Z., Takemura, S.-y., Hayworth, K. J., et al. (2020). A connectome and analysis of the adult *Drosophila* central brain. *Elife* 9, e57443. doi:10.7554/eLife.57443

Xu, C. S., Januszewski, M., Lu, Z., Takemura, S.-y., Hayworth, K., Huang, G., et al. (2020). A connectome of the adult *Drosophila* central brain. *BioRxiv*
